# Knowledge, Attitudes, and Sources of Information on Vaccines in Spanish Nursing Students: A Cross-Sectional Study

**DOI:** 10.3390/ijerph18073356

**Published:** 2021-03-24

**Authors:** José Tuells, Cecilia M. Egoavil, Isabel Morales-Moreno, Elena Fortes-Montoya, Carlos Salazar-García, Noelia Rodríguez-Blanco

**Affiliations:** 1 Department of Community Nursing, Preventive Medicine and Public Health and History of Science, University of Alicante, San Vicente del Raspeig, 03690 Alicante, Spain; elena.fortes@hotmail.com; 2 Unit of Clinical Pharmacology, Alicante University General Hospital, 03010 Alicante, Spain; egoavil_cec@gva.es (C.M.E.); salazargarciacarlos93@gmail.com (C.S.-G.); 3 Department of Nursing, Universidad Católica San Antonio de Murcia (UCAM) Campus de los Jerónimos, N. 135 Guadalupe, 30107 Murcia, Spain; imorales@ucam.edu; 4 Department of Obstetrics and Gynaecology, Marina Baixa University Hospital, Av. Alcalde En Jaume Botella Mayor, 7, 03570 Villajoyosa, Spain; noelia.rodriguez@uchceu.es; 5 Department of Nursing, Universidad Cardenal Herrera-CEU, Plaza Reyes Católicos, 19, 03204 Elche, Spain

**Keywords:** vaccines, students, universities, attitudes, acceptance, nursing, knowledge

## Abstract

Health professionals are the most influential and main sources of information about vaccines for the general population, as they are regarded as role models by patients and society. The objective of the present study was to determine the knowledge and attitudes of a group of university Nursing students about vaccines, as well as their sources of information and their education needs. A cross-sectional study was performed through a questionnaire (55 items) provided to Nursing students at two Spanish universities. A total of 1122 students participated in the study. The mean score obtained for knowledge about vaccines was 44.6 ± 4.3, and for attitudes towards vaccines, it was 37.2 ± 3.9. Hepatitis B (94.7%) and the Flu (89%) are the two main vaccines they should receive as health workers. The main source of information was the family environment (65.6%). Most of them considered that post-graduate education about vaccines should be provided by academic entities (universities, 62.7%). Among the health professionals, Nurses (85.5%) must be better educated and trained on the subject of vaccines. It is therefore necessary to delve into and complete the nurses’ training on vaccines, to educate them about the risks at the individual level, and their decisive role as promoters of the vaccination strategy for the general population. Universities must become the leaders in vaccine education and training.

## 1. Introduction

Health professionals are the most influential and main source of information about vaccines for the general population [1,2,3,4]. A patient’s confidence on her or his nurse and/or doctor has been associated to their final acceptance or rejection of vaccines [5,6]. Despite the education programs of nurses and other professionals, a variability in their knowledge is still found amongst the nurses themselves, so that the design of refresher programs for these professionals could improve their vaccine coverage and their patient’s as well [7].

The WHO argues that to increase vaccine coverage, we should promote vaccination leadership through health professionals as a strategy to improve both vaccine acceptance and coverage, thereby strengthening Primary Care [8]. Increasing vaccination beyond childhood was one of the WHO challenges for 2020, including the vaccination of high-risk groups, among which we find health professionals and health science students [8]. In these two groups, vaccination provides clear benefits for them as a high risk group, and because they can actively promote vaccination in their professional practice [7].

With regards to vaccination, Nursing university students act according to their degree of knowledge and attitudes, which determines their own vaccination practices, and at same time influences the vaccination of their patients [9,10,11,12]. Vaccination as a preventive measure in student groups should not only be considered an individual practice of self-protection, but an act of community disease prevention as well [5,13]. Being an example allows increasing the rates of vaccination in the general population, as health professionals are regarded as role models by patients and society in general.

Education related to vaccines is heterogeneous and diverse. In Spain, this is dependent on the universities, as the design of education programs tend to be flexible, although the final objective is to obtain specific competences that are defined at the state level [12,14]. The management of vaccination interventions is carried out in Spain by Nursing professionals, but collaboratively with the physician, not as an independent intervention, as in other countries such as the UK, where the prescription of pharmacological products by Nurses is recognized and included in the training programs of the different healthcare specialties.

The European Commission and the WHO have classified doubts about vaccines and vaccination in general as the main threats to global health, thereby highlighting the educational needs about vaccines of future professionals [15].

The purpose of this study was to determine the knowledge and attitudes of a group of nursing degree university students from two different Spanish universities, related to vaccines, and their most common sources of information in this area of knowledge.

## 2. Materials and Methods

### 2.1. Design, Population, and Sample

A cross-sectional descriptive study was conducted at two Spanish universities, one public (University of Alicante, UA), and another private (Catholic University of Murcia San Antonio (UCAM)). The study included all the nursing students enrolled in the 4 academic years of the Nursing degree in Spain.

### 2.2. Sampling Method

In the 2018–2019 academic year, there was a total of 2154 nursing students enrolled at both universities, 830 (UA), and 1324 (UCAM). During their regular classes, a questionnaire was distributed until a minimum of 30% of the total number of students was reached. During the 2018–2019 academic year there was a total of 47,229 nursing students in Spain. Our sample represented 4.5% of the total.

### 2.3. Data Collection Tools

An ad hoc questionnaire was utilized as the data collection instrument. It was previously validated at the University of Alicante, and was based on previous studies [16,17,18,19]. Its content was evaluated by the members of the research group, whom, after providing their recommendations, approved the final version in consensus. A pilot study was conducted with 30 health sciences university students (15 men, and 15 women) who were not taken into consideration for the study [20,21]. The questionnaire was anonymous, standardized, and self-administered. Participation was voluntary and without incentives. The members of the research group explained the purpose of the study in each classroom, before providing the questionnaire, which could be completed in 10 min.

The questionnaire included 55 questions divided into six sections:

1. Socio-demographic variables: information on gender, age, nationality, academic year and university, marital status, and number of children, were collected.
2. Knowledge and attitudes about vaccination: a total of 21 items (items 1–12 for knowledge, and items 13–21 for attitudes), organized in a Likert format with 5 response options [20], (“strongly disagree” to “strongly agree”), to indicate the level of agreement with the statements.

The general scale showed an acceptable level of reliability (Cronbach’s alpha = 0.743), consistent with other studies [22], with the result for the knowledge section being 0.647, and for attitudes, 0.664. The cutoff point was established on the basis of ±1 SD.

The matrix of correlations test between the items revealed the predominance of correlations higher than 0.1 between the items that supported the suitability for factorization. Additionally, a factor analysis of structural validity was applied, based on the value of the Kaiser-Meyer-Olkin test (KMO = 0.88), as well as Bartlett’s sphericity test (χ^2^ = 5580.79, df = 190, *p* < 0.01). An exploratory factorial analysis was performed, utilizing the factorization of the main components with Varimax rotation. The results provided 4 factors that represented 53.3% of variance of the item (factor values 30.1, 8.9, 7.7, and 6.1), as shown in Table 1.

3. Knowledge of vaccines of the health professionals: this section evaluated the knowledge of the students in regard to the vaccines that are necessary for health professionals for their profession. They were asked to state which of the vaccines were necessary for this group of professionals, from a list of 16 pathogens for which a vaccine was available. Sixteen items were answered with a simple “true/false”, and the option “I don’t know”. The scores for this knowledge oscillated between 1 and 16, and points were awarded for the correct responses.
4. Sources of information: the participants were asked to point out their main sources of information on vaccines (items 38–49), and the number of classes throughout their studies where they had received information about immunization (item 50).
5. Negative experiences with vaccines: they were asked to find out, in their immediate environment, if anyone had had a negative experience after receiving a vaccine (items 51–53).
6. Opinion about the teaching of vaccinology: in the final section of the questionnaire, the student had to provide an opinion about which health professionals should be better educated about vaccines, and likewise, to choose an institution which should have a greater responsibility for post-graduate education and training related to vaccines (items 54–55) [16].

### 2.4. Methods of Analysis

Descriptive statistics were utilized to present the characteristics of the sample, thus, the means and standard deviations (SD) were calculated. We utilized a Student’s t test to compare the age of the students, and Chi-square tests to examine the differences as a function of the type of university (UA, UCAM), and the level of education (Lower = 1st–2nd year, Upper = 3rd–4th year) in all the items of the questionnaire. Additionally, the OR of each item and the 95%CI were calculated, by comparing a specific answer with the rest of them. The statistics tests utilized a one-way ANOVA to establish statistically-significant groups, mean differences in the scores of the subscales of knowledge and attitude, for the categorical variables selected: university, level of education. Poor or good knowledge were considered if a score 2SD from the mean was obtained for each of the questions.

All the analyses were performed with the statistical program SPSS Statistics for Windows v20 (SPSS v20, IBM Corp., Armonk, NY, USA). The level of accepted statistical significance was *p* < 0.05.

### 2.5. Ethical Considerations

All subjects gave their informed consent agreement for participation. The study is compliant with the UA University Ethical Committee Standards. The study is in accordance with the Helsinki Declaration and EU Regulation 134 2016/679 (GDPR) concerning the processing of personal data. Participation was completely voluntary, and all students were asked to provide their written informed consent and to sign the top of the survey before participating. Participants were informed that all the information collected would be anonymous and would be treated as confidential. Participants cannot be identified from the material.

## 3. Results

### 3.1. Population Description

From a total of 2154 students enrolled in the Nursing Degree at both universities (830 UA and 1324 UCAM), the participation of 1498 students was obtained, for a total rate of 69.5% (1498/2154). A total of 1122 participants accessed the questionnaire (total response rate of 52.1% (1122/2154)), with a distribution according to university of 86.2% (716/830) for the UA, and 30.6% (406/1324) for the UCAM.

Among the participants, 296 (26.3%) were 1st year students, 317 (28.2%) were 2nd year students, and 286 (25.4%) and 223 (19.9%) were 3rd and 4th year students, respectively. Additionally, 904 (80.6%) were women (women/men ratio 4:1), and the mean age (±SD) was 21.4 ± 4.7 years old. Most were single and without children, and their country of origin was Spain (Table 2).

### 3.2. Knowledge and Attitudes of Nurse Students toward Vaccination

The mean score obtained for knowledge about vaccines was 44.6 ± 4.3, and for attitude about the vaccines, this was 37.2 ± 3.9, indicating positive qualities overall, with the students having a good level of knowledge and attitudes. Significant differences were not found according to gender, marital status, or number of children. Knowledge and attitudes were affected by the type of university at which the students were enrolled [OR 2.9, 95%CI (2.1–3.9)], and [OR 2.1 95%CI (1.5–2.9)], respectively, the number of years studied [OR 4.0 95%CI (2.8–5.8)] and [OR 1.7 95%CI (1.3–2.2)], and having taken a specific class where the vaccine-related subjects were taught [OR 2.2 95%CI (1.5–3.1)] and [OR 1.7 95%CI (1.1–2.6)]. Lastly, 27.3% (3017/1121) of the students who completed the questionnaire indicated having some type of negative reference about vaccination from a person close to them (Table 3).

Table 4 (knowledge) and Table 5 (attitudes) show the details of the questions from the questionnaire, with statistically significant differences found in all the items according to type of university and academic years.

Most of those polled, 98.0% (1100/1122), considered that the vaccines prevented diseases, that they were safe, 88.7% (972/1122), and that they had eradicated diseases, 91.5% (1026/1122). Additionally, they considered them as beneficial for children, 96.4% (1081/1122), the child’s surroundings, 93.5% (1049/1122), and that they should be mandatory, 79.9% (897/1122). Similarly, 93.0% (1044/1122) believed that health sciences students should be vaccinated during their pre-professional practices, although only 43.7% (491/1122) knew the vaccination schedule. Furthermore, 29.8% (334/1122) had a neutral opinion on the supposed severe secondary effects such as autism, and their indication, or not, for pregnant women, 44.8% (502/1122). Only 29.4% (329/1122) of those polled considered that they had received enough information about vaccines, with significant different observed according to the universities (Table 4).

As for attitudes (Table 5), only 16.1% (181/1122) considered the vaccine information provided to the general public as adequate; 95.6% (1073/1122) declared having a favorable opinion towards vaccines, 95.3% (1069/1122) would recommend vaccination to their future patients, and 93.9% (1054/1122) believed that the professionals in health centers should be vaccinated. Those polled considered that health science students should be vaccinated against hepatitis B, 92.3% (1036/1122), the flu, 83.4% (936/1122), and that men should be vaccinated against HPV 73.3% (822/1122), although a small percentage was also found who had doubts about the flu vaccine (13.2%), and HPV (16.4%). Question 18 assessed whether or not there was a difference in opinion according to the sex of students, with the differences found to be non-significant. Additionally, 75.2% (844/1122) considered that the vaccine against meningococcus B should be included in the vaccination schedule, but with an important percentage of doubt (20%). Lastly, 61.8% (694/1122) had the opinion that their university study plan should dedicate more class hours to vaccine-related education, although 30.9% of those polled were indifferent about this aspect.

A final question in this section inquired about the administration of vaccines at the prevention or assistance services of the university, with only 5.2% (58/1122) knowing about its existence, and 3.5% (39/1122) using these services at some point.

### 3.3. Knowledge Regarding Healthcare Workers Vaccination

Table 6 summarizes the results with respect to the knowledge about the vaccines recommended for health professionals (16 items). Of those polled, 44.1% (495/1122) obtained low scores with 10 or less correct answers, and only 34.6% (388/1122) obtained more than 14 correct answers. The table shows the distribution of the mean according to the variables studied.

**Figure 1:**
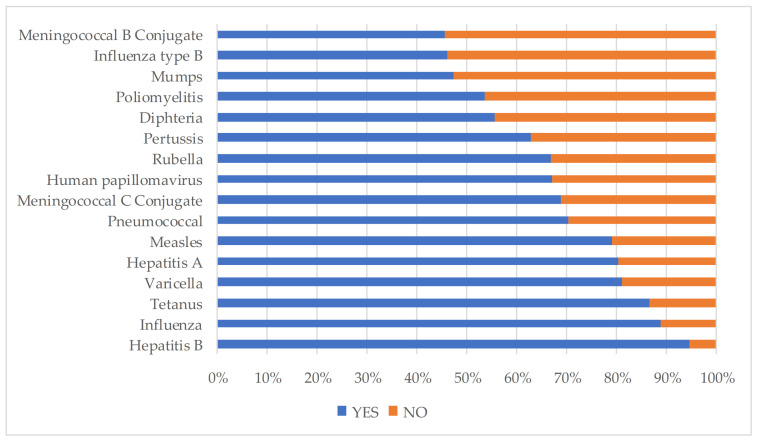
Knowledge about the vaccines that are necessary for health professionals.

The distribution of the knowledge about vaccination of the health professionals for each vaccine, are shown in Figure 1. The vaccines against Hepatitis B and Influenza were scored higher by the nursing students, and more than 50% of the students considered that vaccination against Meningococcus, Haemophilus type b, or mumps, was not necessary for health workers.

### 3.4. Sources of Information

**Figure 2:**
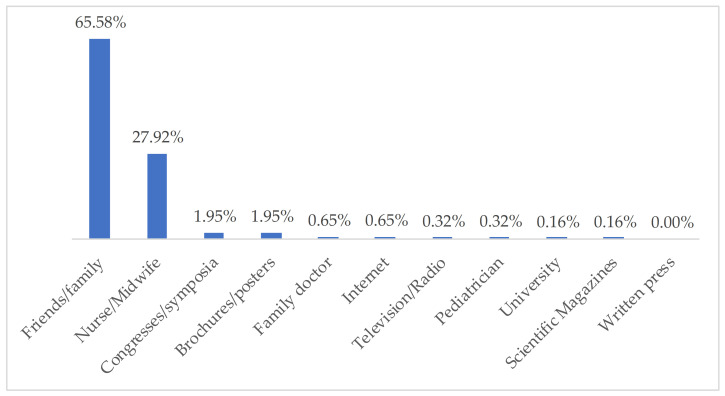
Sources of information about vaccines.

The main sources of information about vaccines mentioned by the nursing students from both universities came from the family surroundings, 65.6% (61.8% UA, and 71.3% UCAM), and from the work/university environment, 27.9% (nursing personnel, 29.5% UA, and 25.4% UCAM). Other professionals mentioned were family doctors and pediatricians, although for only 1%, and the sources of scientific and written information were little represented (Figure 2).

### 3.5. Opinion on Post-Graduate Training in Vaccinology

**Figure 3:**
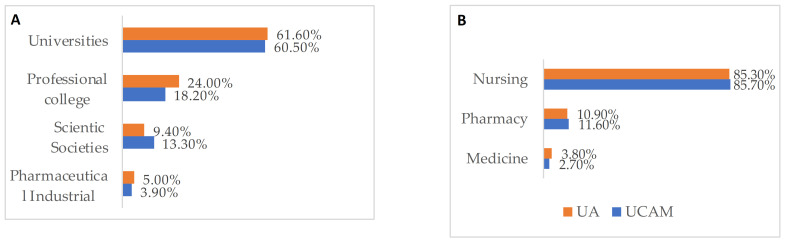
Opinion about who should receive vaccine-related education (**A**) from who should provide it (**B**).

Figure 3 shows that most of the nursing students considered that the post-graduate education about vaccines should be the responsibility of academic entities, such as universities, 62.7% (703/1122), and professional schools, 21.9% (246/1122). Likewise, those polled had the opinion that the health personnel who should be better educated and have more information about vaccine-related subjects, with nurses in first place, 85.5% (595/1122), followed by doctors, 11.1% (125/1122), and pharmacists, 3.4% (38/1122).

## 4. Discussion

The interaction between the patient and the health personnel is fundamental for maintaining trust on vaccines. Various studies have pointed out that the knowledge and attitudes of health professionals related to vaccines are decisive factors for their own immunization, their intention of recommending the vaccine to their patients, and the patient’s acceptance of vaccines [23,24,25].

The nursing students in our study had a good knowledge about vaccines, independently of the type of university (public or private). However, interesting nuances were found when assessing the strength of their opinions, with the students from the UA showing more trust and less doubt, with a greater ratio of 1 and 5 values in the Likert scale. The explanation for these differences requires a more in-depth study on the teaching programs and the teaching methodology utilized, which are not the subject of the present study. However, the recent literature shows that education programs can be useful for disseminating knowledge and correcting information that is destined towards vaccination adherence [26,27,28,29].

Although most (93%) of the students considered that they should be vaccinated before their pre-professional practices, a great variability about the types of vaccines needed was found. Thus, while the flu vaccine was accepted by 83.4%, the vaccines against hepatitis B obtained a figure of 92%. This discrepancy in the attitudes and behavioral practices could be associated to the variation of the individual decisions according to the sociocultural context, the social circumstances, and the personal experience [23,30,31]. They were also doubtful about the secondary effects and the vaccines recommended for their professional development.

The students were in agreement with mandatory vaccination and the importance of their professional status as vaccination promoters for the immunization of the population [32], but knowledge about the pathogens and their vaccines was irregular, with the ones associated to work hazards being more recognized, such as Hepatitis B (94.7%) and the Flu (89.0%), as compared to other pathogens such as Meningitis (45.5%), or Haemophilus Influenzae Type b (46.0%). These differences in knowledge have been previously described in health professionals with a similar distribution, in a poll conducted by Maltezou et al. [33], where 90% of the health workers identified the Hepatitis B vaccine as recommended, and only 26% did so for Hepatitis A, and the study by Tamburrano et al. [7], which described low percentages for Hepatitis A (22%) and Meningococcus (41%), as well as high percentages for Hepatitis B (92%).

The percentage of students who had a favorable opinion towards vaccines, and who would recommend them to their future patients and health professionals was higher than 93%, a higher percentage than other studies with health professionals [34,35,36]. Additionally, they were in agreement with lengthening the vaccination schedule and including men in the vaccine against HPV.

It was also found that the nursing students identified themselves as future relevant actors in the process of vaccination and promotion of health, when considering that they were the ones who should be better educated on the subject matter. They also showed a critical opinion on the inadequate information received by the population in general about vaccines (84%), which they considered it to be scarce. Their opinion was similar in regard to their university education; the students in the upper academic years believed that the teaching load on the subject was insufficient, and therefore thought that more class hours should be dedicated to this in the study plan. This information indicates that the students demand more education that is more specific to vaccines and vaccination-related subjects. Thus, it is interesting to point out that aside from the more specific technical aspects of the subject, communication is still a fundamental requisite in the education of these health professionals.

We also found that the main sources of information of the nursing students were their close environments, such as friends and family, followed by the nursing personnel and midwives, with the communication media being the least consulted. These results show the importance of the social environment in increasing the confidence and acceptability of the vaccines among the nursing students, and also shows an opposite behavior to that found in other population groups. For example it was found that essential workers utilized communication media as the main sources of information [37]. Thus, we believe that education pre- and post-university should be enforced, even in places with a high level of competence related to vaccines, where we find the Nursing students, to improve the current rates of vaccine coverage of the students, as well as the health professionals in general [38,39]. More extensive training in the last years of a university career in nursing and continuous training of active professionals would be necessary, since epidemiology evolves, changes are created in the vaccination schedule, and new vaccines appear, such as SARS-CoV-2, which require constant updating.

Extracurricular activities and continuous training could be a positive predictor for vaccination [39,40], and the students in the present study believed that the academic institutions (universities, professionals schools) should be responsible for post-graduate vaccine-related education, as compared to scientific societies and the pharmaceutical industry.

We believe that there is a need to create a comprehensive vaccination service for Health Sciences students at the regional level, which could offer counseling and care services to the students from different universities and other health-related degrees.

Universities as a whole must generate a positive impact on the attitudes about vaccines of the nursing students through an improvement in education, with vaccination being an essential practice for the self-care of the students. This should be regarded as a key activity for strengthening their role as health promoters, having in mind that in vaccination programs in Spain, it is the nursing personnel who are responsible for their management.

The main limitation of the study is that we did not take into account the education programs of the universities that participated in the study, which could be utilized to identify the needs of improvement at the educational level associated with the education programs implemented. Additionally, the vaccination status of the students was not taken into account, and this could have an impact on their future vaccination as health professionals.

## 5. Conclusions

The nursing students had positive attitudes towards vaccines, with a good level of knowledge, especially the students in the upper academic years. The predominant reliable source of information for this group of students was the social/family environment, followed by nurses. It is necessary to complete their education to make the nursing students aware about the level of individual risk and their decisive role as promoters of the vaccination strategy of the general population. The results of the present study suggest that it could be beneficial to provide additional information directed to the nursing students, to clarify their worries about vaccine safety and to help improve the acceptance of vaccines in the communities cared for by these future health professionals. The post-graduate vaccine-related education should be associated to academic institutions.

## Figures and Tables

**Table 1:** Knowledge and attitudes about vaccines.

Items	Mean	SD	Corrected Item-Total Correlation	Cronbach’s If Item Deleted
A01	4.78	0.487	0.492	0.728
A02	4.21	0.718	0.435	0.725
A03	4.56	0.748	0.381	0.728
A04	4.39	0.653	0.497	0.723
A05	4.68	0.571	0.564	0.721
A06	4.64	0.665	0.556	0.719
A07	3.23	1.278	0.297	0.737
A08	4.66	0.675	0.494	0.722
A09	2.11	1.071	-0.177	0.776
A10	2.67	1.043	-0.119	0.770
A11	2.84	1.168	0.128	0.752
A12	4.16	0.925	0.455	0.721
A13	2.47	1.003	0.108	0.750
A14	4.63	0.650	0.514	0.722
A15	4.65	0.611	0.490	0.724
A16	4.62	0.634	0.553	0.720
A17	3.74	0.922	0.200	0.742
A18	3.98	1.166	0.237	0.741
A19	4.34	0.870	0.453	0.722
A20	4.62	0.669	0.543	0.720
A21	4.17	0.970	0.339	0.730

**Table 2:** Sample characteristics (*n* = 1498).

Characteristic	UA	UCAM	Total	OR	*p*-Value
	*n* (%)	*n* (%)	*n* (%)	(95%CI)	
Academic year					
Lower	357 (49.9)	256 (63.1)	613 (54.6)	1.7 (1.3–2.2)	<0.001
Upper	359 (50.1)	150 (36.9)	509 (45.4)		
Gender					
Men	124 (17.3)	94 (23.2)	218 (19.4)	1.4 (1.1–1.9)	0.018
Women	592 (82.7)	312 (76.8)	904 (80.6)		
Marital status					
Single	694 (96.9)	389 (95.8)	1083 (96.5)	0.7 (0.4–1.4)	NS
Couple	22 (3.1)	17 (4.2)	39 (3.8)		
Number of Children					
None	693 (96.8)	397 (97.8)	1090 (97.2)	1.5 (0.7–3.2)	NS
One or more	23 (3.2)	9 (2.2)	32 (2.9)		
Country of Origin (*n* = 1119)					
Spain	690 (96.8)	396 (97.5)	1086 (96.8)	1.3 (0.6–2.8)	NS
Others	23 (3.2)	10 (2.5)	33 (2.9)		
Mean age (SD)	21.1 (4.9)	21.9 (4.2)	21.4 (4.7)		0.005

NS: Not significant; UA: University of Alicante; UCAM: Catholic University of Murcia San Antonio; CI: Confidence Interval.

**Table 3:** Knowledge and attitude scores associated to demographics and population characteristics.

Characteristics	Knowledge				Attitude			
	Poor	Good	OR	*p*-Value	Poor	Good	OR	*p*-Value
	*n* (%)	*n* (%)	(95%CI)		*n* (%)	*n* (%)	(95%CI)	
Gender								
Men	42 (20.6)	176 (19.2)	1.1 (0.7–1.6)	NS	169 (77.5)	49 (22.5)	1.1 (0.7–1.5)	NS
Women	162 (79.4)	741 (80.8)			692 (76.6)	211 (23.4)		
Academic year								
Lower	162 (79.4)	450 (49.1)	4 (2.8–5.8)	<0.01	496 (81.0)	116 (19.0)	1.7 (1.3–2.2)	<0.001
Upper	42 (20.6)	467 (50.9)			365 (71.7)	144 (28.3)		
Marital status								
Single	198 (97.1)	884 (96.4)	1.2 (0.5–3.0)	NS	832 (76.9)	250 (23.1)	1.1 (0.6–2.4)	NS
Couple	6 (2.9)	33 (3.6)			29 (74.4)	10 (25.6)		
Children								
Yes	198 (97.1)	891 (97.2)	1 (0.4–2.4)	NS	839 (77.0)	250 (23.0)	1.5 (0.7–3.3)	NS
No	6 (2.9)	26 (2.8)			22 (68.8)	10 (31.3)		
Nationality								
Spain	197 (96.6)	889 (97.3)	0.8 (0.3–1.9)	NS	833 (97.1)	252 (96.9)	1.1 (0.5–2.4)	NS
Others	7 (3.4)	25 (2.7)			25 (2.9)	8 (3.1)		
Universities								
UA	116 (56.9)	290 (31.6)	2.9 (2.1–3.9)	<0.001	344 (40.0)	62 (23.8)	2.1 (1.5–2.9)	<0.001
UCAM	88 (43.1)	627 (68.4)			517 (60.0)	198 (76.2)		
Knowing someone with negative experience								
Yes	62 (30.4)	245 (26.7)	1.2 (0.9–1.7)	NS	235 (76.5)	72 (23.5)	1 (0.7–1.3)	NS
No	142 (69.6)	672 (73.3)			626 (76.9)	188 (23.1)		
Number of courses with vaccines topic								
No courses	51 (25.0)	122 (13.3)	2.2 (1.5–3.1)	<0.001	146 (83.9)	28 (16.1)	1.7 (1.1–2.6)	0.016
1 or + courses	153 (75.0)	795 (86.7)			715 (75.5)	232 (24.5)		

NS: Not significant; UA: University of Alicante; UCAM: Catholic University of Murcia San Antonio; CI: Confidence Interval.

**Table 4:** Vaccine knowledge questionnaire score distribution.

Items	University				Academic Year			
	UA *n* (%)	UCAM *n* (%)	OR (95%CI)	*p*-Value	Lower	Upper	OR (95%CI)	*p*-Value
**1. Vaccines are useful for preventing diseases.**								
Strongly disagree	0 (0.0)	2 (0.5)	null		1 (0.2)	1 (0.2)	0.8 (0.1–13.3)	
Disagree	1 (0.1)	1 (0.2)	1.8 (0.1–28.3)		1 (0.2)	1 (0.2)	0.8 (0.1–13.3)	
Neutral	5 (0.7)	13 (3.2)	4.7 (1.7–13.3)	<0.001	16 (2.6)	2 (0.4)	6.8 (1.6–29.7)	<0.001
Agree	83 (11.6)	119 (29.3)	3.2 (2.3–4.3)		124 (20.2)	78 (15.3)	1.4 (1.0–1.9)	
Strongly agree	627 (87.6)	271 (66.7)	0.3 (0.2–0.4)		471 (76.8)	427 (83.9)	0.6 (0.5–0.9)	
**2. Vaccines are safe.**								
Strongly disagree	1 (0.1)	3 (0.7)	5.3 (0.6–51.3)		2 (0.3)	2 (0.4)	0.8 (0.1–5.9)	
Disagree	3 (0.4)	10 (2.5)	6 (1.6–21.9)		10 (1.6)	3 (0.6)	2.8 (0.8–10.2)	
Neutral	53 (7.4)	80 (19.7)	4.7 (1.7–13.3)	<0.001	99 (16.2)	34 (6.7)	2.7 (1.8–4.1)	<0.001
Agree	340 (4.5)	224 (55.2)	1.4 (1.1–1.7)		325 (53.0)	239 (47.0)	1.3 (1.0–1.6)	
Strongly agree	319 (44.6)	89 (21.9)	0.3 (0.3–0.5)		177 (28.9)	231 (45.4)	0.5 (0.4–0.6)	
**3. Vaccines have been able to eradicate diseases.**								
Strongly disagree	0 (0.0)	7 (1.7)	null		1 (0.2)	6 (1.2)	0.1 (0.0–1.1)	
Disagree	7 (1.0)	15 (3.7)	3.9 (1.6–9.6)		16 (2.6)	6 (1.2)	2.2 (0.9–5.8)	
Neutral	25 (3.5)	42 (10.3)	3.2 (1.9–5.3)	<0.001	43 (7.0)	24 (4.7)	1.5 (0.9–2.5)	<0.001
Agree	137 (19.1)	125 (30.8)	1.9 (1.4–2.5)		172 (28.1)	90 (17.7)	1.8 (1.4–2.4)	
Strongly agree	547 (76.4)	217 (53.4)	2.8 (2.2–3.7)		381 (62.2)	383 (75.2)	1.9 (1.4–2.4)	
**4. Vaccines are effective.**								
Strongly disagree	0 (0.0)	2 (0.5)	null		1 (0.2)	1 (0.2)	0.8 (0.1–13.3)	
Disagree	2 (0.3)	4 (1.0)	3.6 (0.6–19.5)		5 (0.8)	1 (0.2)	4.2 (0.5–35.9)	
Neutral	24 (3.4)	52 (12.8)	4.2 (2.6–7.0)	<0.001	56 (9.1)	20 (3.9)	2.5 (1.5–4.2)	<0.001
Agree	287 (40.1)	216 (53.2)	1.7 (1.3–2.2)		305 (49.8)	198 (38.9)	1.6 (1.2–2.0)	
Strongly agree	403 (56.3)	132 (32.5)	0.4 (0.3–0.5)		246 (40.1)	289 (56.8)	0.5 (0.4–0.6)	
**5.Vaccinating a child is a health benefit.**								
Strongly disagree	0 (0.0)	3 (0.7)	null		2 (0.3)	1 (0.2)	1.7 (0.2–18.4)	
Disagree	1 (0.1)	2 (0.5)	3.5 (0.3–39.2)		2 (0.3)	1 (0.2)	1.7 (0.2–18.4)	
Neutral	13 (1.8)	22 (5.4)	3.1 (1.5–6.2)	<0.001	26 (4.2)	9 (1.8)	2.5 (1.1–5.3)	<0.001
Agree	125 (17.5)	145 (35.7)	2.6 (2.0–3.5)		179 (29.2)	91 (17.9)	1.9 (1.4–2.5)	
Strongly agree	577 (80.6)	234 (57.6)	0.3 (0.3–0.4)		404 (65.9)	407 (80.0)	0.5 (0.4–0.6)	
**6. Vaccinating a child is a benefit for his/her environment.**								
Strongly disagree	1 (0.1)	3 (0.7)	5.3 (0.6–51.3)		3 (0.5)	1 (0.2)	2.5 (0.3–24.1)	
Disagree	7 (1.0)	6 (1.5)	1.5 (0.5–4.6)		12 (2.0)	1 (0.2)	10.1 (1.3–78.3)	
Neutral	25 (3.5)	31 (7.6)	2.3 (1.3–3.9)	<0.001	50 (8.2)	6 (1.2)	7.4 (3.2–17.5)	<0.001
Agree	106 (14.8)	134 (33.0)	2.8 (2.1–3.8)		151 (24.6)	89 (17.5)	1.5 (1.2–2.1)	
Strongly agree	577 (80.6)	232 (57.1)	0.3 (0.2–0.4)		397 (64.8)	412 (80.9)	0.4 (0.3–0.6)	
**7. I know the vaccination schedule of my autonomous community.**								
Strongly disagree	79 (11.0)	61 (15.0)	1.4 (1–2)		127 (20.7)	13 (2.6)	10.0 (5.6–17.9)	
Disagree	111 (15.5)	66 (16.3)	1.1 (0.8–1.5)		134 (21.9)	43 (8.4)	3.0 (2.1–4.4)	
Neutral	218 (30.4)	96 (23.6)	0.7 (0.5–0.9)	<0.001	203 (33.1)	111 (21.8)	1.8 (1.4–2.3)	<0.001
Agree	209 (29.2)	63 (15.5)	0.4 (0.3–0.6)		94 (15.3)	178 (35.0)	0.3 (0.3–0.4)	
Strongly agree	99 (13.8)	120 (29.6)	2.6 (1.9–3.5)		55 (9.0)	164 (32.2)	0.2 (0.1–0.3)	
**8. It is important for the health sciences students to be vaccinated to avoid the transmission of infectious diseases during their clinical practices.**								
Strongly disagree	1 (0.1)	6 (1.5)	10.7 (1.3–89.4)		5 (0.8)	2 (0.4)	2.1 (0.4–10.8)	
Disagree	5 (0.7)	3 (0.7)	1.1 (0.3–4.5)		6 (1.0)	2 (0.4)	2.5 (0.5–12.5)	
Neutral	31 (4.3)	32 (7.9)	1.9 (1.1–3.1)	<0.001	39 (6.4)	24 (4.7)	1.4 (0.8–2.3)	<0.001
Agree	117 (16.3)	89 (21.9)	1.4 (1.1–2.0)		123 (20.1)	83 (16.3)	1.3 (0.9–1.8)	
Strongly agree	562 (78.5)	276 (68.0)	0.6 (0.4–0.8)		440 (71.8)	398 (78.2)	0.7 (0.5–0.9)	
**9. Vaccines can cause diseases such as autism or multiple sclerosis.**								
Strongly disagree	310 (43.3)	120 (29.6)	0.5 (0.4–0.7)		176 (28.8)	254 (49.9)	0.4 (0.3–0.5)	
Disagree	176 (24.6)	82 (20.2)	0.8 (0.6–1.0)		139 (22.7)	119 (23.4)	1.0 (0.7–1.3)	
Neutral	200 (27.9)	134 (33.0)	1.3 (1.0–1.7)	<0.001	238 (38.9)	96 (18,9)	2.7 (2.1–3.6)	<0.001
Agree	21 (2.9)	50 (12.3)	4.6 (2.7–7.8)		46 (7.5)	25 (4.9)	1.6 (1.0–2.6)	
Strongly agree	8 (1.1)	20 (4.9)	4.6 (2.0–10.5)		13 (2.1)	15 (2.9)	0.7 (0.3–1.5)	
**10. Vaccines are contraindicated for pregnant women.**								
Strongly disagree	160 (22.3)	27 (6.7)	0.2 (0.2–0.4)		56 (9.2)	131 (25.7)	0.3 (0.2–0.4)	
Disagree	170 (23.7)	70 (17.2)	0.7 (0.5–0.9)		115 (18.8)	125 (24.6)	0.7 (0.5–0.9)	
Neutral	291 (40.6)	211 (5.02)	1.6 (1.2–2.0)	<0.001	315 (51.5)	187 (36.7)	1.8 (1.4–2.3)	<0.001
Agree	67 (9.4)	72 (17.7)	2.1 (1.5–3.0)		95 (15.5)	44 (8.6)	1.9 (1.3–2.8)	
Strongly agree	27 (3.8)	26 (6.4)	1.7 (1.0–3.0)		31 (5.1)	22 (4.3)	1.2 (0.7–2.1)	
**11. The education I have received during my degree about vaccines is enough.**								
Strongly disagree	107 (14.9)	43 (10.6)	0.7 (0.5–1.0)		109 (17.8)	41 (8.1)	2.5 (1.7–3.6)	
Disagree	224 (31.3)	86 (21.2)	0.6 (0.4–0.8)		170 (27.7)	140 (27.5)	1.0 (0.8–1.3)	
Neutral	237 (33.1)	96 (23.6)	0.6 (0.5–0.8)	<0.001	209 (34.1)	124 (24.4)	1.6 (1.2–2.1)	<0.001
Agree	127 (17.7)	97 (23.9)	1.5 (1.1–2.0)		96 (15.7)	128 (25.1)	0.6 (0.4–0.7)	
Strongly agree	21 (2.9)	84 (20.7)	8.6 (5.3–14.2)		29 (4.7)	76 (14.9)	0.3 (0.2–0.4)	
**12. Vaccination should be mandatory to achieve universal coverage.**								
Strongly disagree	11 (1.5)	9 (2.2)	1.5 (0.6–3.5)		12 (2.0)	8 (1.6)	1.3 (0.5–3.1)	
Disagree	25 (3.5)	16 (3.9)	1.1 (0.6–2.1)		29 (4.7)	12 (2.4)	2.1 (1.0–4.1)	
Neutral	97 (13.5)	67 (16.5)	1.3 (0.9–1.8)	0.005	103 (16.8)	61 (12.0)	1.5 (1.1–2.1)	0.004
Agree	247 (34.5)	171 (42.1)	1.4 (1.1–1.8)		239 (39.0)	179 (35.2)	1.2 (0.9–1.5)	
Strongly agree	336 (46.9)	143 (35.2)	0.6 (0.5–0.8)		230 (37.5)	249 (48.9)	0.6 (0.5–0.8)	

UA: University of Alicante; UCAM: Catholic University of Murcia San Antonio; OR is calculated for each response against all the others. *p*-value: calculated for the group, chi-square test; CI: Confidence Interval.

**Table 5:** Vaccine attitude questionnaire score distribution.

Items	University				Course			
	UA *n* (%)	UCAM *n* (%)	OR (95%CI)	*p*-Value	First Years	Older Years	OR (95%CI)	*p*-Value
**13. The information received by the population is adequate.**								
Strongly disagree	106 (15.5)	56 (15.8)	0.9 (0.6–1.3)		102 (16.6)	60 (11.8)	1.5 (1.1–2.1)	
Disagree	338 (41.1)	154 (42.5)	0.7 (0.5–0.9)		277 (45.2)	215 (42.2)	1.1 (0.9–1.4)	
Neutral	189 (28.0)	98 (26.4)	0.9 (0.7–1.2)	<0.001	139 (22.7)	148 (29.1)	0.7 (0.5–0.9)	NS
Agree	68 (11.8)	73 (11.6)	2.1 (1.5–3.0)		74 (12.1)	67 (13.2)	0.9 (0.6–1.3)	
Strongly agree	15 (3.6)	25 (3.6)	3.1 (1.6–5.9)		21 (3.4)	19 (3.7)	0.9 (0.5–1.7)	
**14. The health professionals in health centers should be vaccinated.**								
Strongly disagree	2 (50.0)	3 (50.0)	2.7 (0.4–16.0)		4 (0.7)	1 (0.2)	3.3 (0.4–29.9)	
Disagree	0 (33.3)	6 (66.7)	null		5 (0.8)	1 (0.2)	4.2 (0.5–35.9)	
Neutral	30 (27.5)	27 (72.5)	1.6 (1.0–2.8)	<0.001	28 (4.6)	29 (5.7)	0.8 (0.5–1.4)	<0.001
Agree	157 (31.9)	112 (68.1)	1.4 (1.0–1.8)		162 (26.4)	107 (21.0)	1.3 (1.0–1.8)	
Strongly agree	527 (32.8)	258 (67.2)	0.6 (0.5–0.8)		414 (67.5)	371 (72.9)	0.8 (0.6–1.0)	
**15. I will recommend and foment the vaccines needed in my future patients.**								
Strongly disagree	0 (0.3)	2 (0.2)	null		2 (0.3)	0 (0.0)	null	
Disagree	2 (0.3)	7 (0.2)	6.3 (1.3–30.3)		4 (0.7)	5 (1.0)	0.7 (0.2–2.5)	
Neutral	23 (2.6)	19 (2.8)	1.5 (0.8–2.8)	<0.001	18 (2.9)	24 (4.7)	0.6 (0.3–1.1)	NS
Agree	136 (16.1)	140 (17.3)	2.2 (1.7–3)		167 (27.2)	109 (21.4)	1.4 (1.0–1.8)	
Strongly agree	555 (80.6)	238 (79.6)	0.4 (0.3–0.5)		422 (68.8)	371 (72.9)	0.8 (0.6–1.1)	
**16. My opinion on vaccines is favorable in general.**								
Strongly disagree	0 (0.7)	2 (0.2)	8.9 (1.0–76.6)		4 (0.7)	2 (0.4)	1.7 (0.3–9.1)	
Disagree	2 (0.3)	7 (0.3)	2.4 (0.5–10.6)		6 (1.0)	1 (0.2)	5 (0.6–41.8)	
Neutral	23 (2.6)	19 (3.0)	3.2 (1.6–6.5)	<0.001	24 (3.9)	12 (2.4)	1.7 (0.8–3.4)	NS
Agree	136 (17.4)	140 (20.6)	2.5 (1.9–3.2)		193 (31.5)	118 (23.2)	1.5 (1.2–2.0)	
Strongly agree	555 (78.9)	238 (76.0)	0.3 (0.3–0.4)		386 (63.0)	376 (73.9)	0.6 (0.5–0.8)	
**17. I believe that my study plan should dedicate more class hours to vaccines.**								
Strongly disagree	6 (2.3)	19 (3.0)	5.8 (2.3–14.7)		7 (1.1)	18 (3.5)	0.3 (0.1–0.8)	
Disagree	22 (10.5)	34 (8.4)	2.9 (1.7–5.0)		22 (3.6)	34 (6.7)	0.5 (0.3–0.9)	
Neutral	216 (35.1)	131 (34.3)	1.1 (0.8–1.4)	<0.001	227 (37.0)	120 (23.6)	1.9 (1.5–2.5)	<0.001
Agree	305 (36.1)	153 (40.0)	0.8 (0.6–1)		247 (40.3)	211 (41.5)	1.0 (0.8–1.2)	
Strongly agree	167 (16.1)	69 (14.3)	0.7 (0.5–0.9)		110 (17.9)	126 (24.8)	0.7 (0.5–0.9)	
**18. Men should be vaccinated against HPV.**								
Strongly disagree	24 (7.3)	56 (5.3)	4.6 (2.8–7.6)		29 (4.7)	51 (10.0)	0.4 (0.3–0.7)	
Disagree	15 (7.3)	20 (5.5)	2.4 (1.2–4.8)		7 (1.1)	28 (5.5)	0.2 (0.1–0.5)	
Neutral	107 (18.9)	77 (18.8)	1.3 (1.0–1.8)	<0.001	122 (19.9)	62 (12.2)	1.8 (1.3–2.5)	<0.006
Agree	213 (29.8)	132 (28.7)	1.1 (0.9–1.5)		199 (32.5)	146 (28.7)	1.2 (0.9–1.5)	
Strongly agree	356 (36.8)	121 (41.3)	0.4 (0.3–0.6)		255 (41.6)	222 (43.6)	0.9 (0.7–1.2)	
**19. Health sciences students should be vaccinated against the flu.**								
Strongly disagree	3 (3.3)	9 (2.3)	5.4 (1.5–20.0)		8 (1.3)	4 (0.8)	1.7 (0.5–5.6)	
Disagree	12 (4,3)	14 (3.4)	2.1 (1.0–4.6)		18 (2.9)	8 (1.6)	1.9 (0.8–4.4)	
Neutral	91 (12,5)	57 (18,2)	1.1 (0.8–1.6)	<0.001	91 (14.8)	57 (11.2)	1.4 (1.0–2.0)	<0.001
Agree	179 (28,5)	136 (20,9)	1.4 (1.1–1.7)		184 (30.0)	131 (25.7)	1.3 (1.0–1.6)	
Strongly agree	431 (51,5)	190 (55,0)	0.6 (0.5–0.7)		312 (50.9)	309 (60.7)	0.7 (0.5–0.9)	
**20. Health sciences students should be vaccinated against Hepatitis B.**								
Strongly disagree	0 (1.3)	4 (0.6)	null		3 (0.5)	1 (0.2)	2.5 (0.3–24.1)	
Disagree	3 (2.3)	3 (1.6)	1.8 (0.4–8.8)		3 (0.5)	3 (0.6)	0.8 (0.2–4.1)	
Neutral	38 (12.5)	38 (15.5)	1.8 (1.2–2.9)	<0.001	56 (9.1)	20 (3.9)	2.5 (1.5–4.2)	<0.001
Agree	121 (29.2)	123 (20.5)	2.1 (1.6–2.9)		159 (25.9)	85 (16.7)	1.7 (1.3–2.3)	
Strongly agree	554 (54.4)	238 (61.8)	0.4 (0.3–0.5)		392 (63.9)	400 (78.6)	0.5 (0.4–0.6)	
**21. The vaccine against meningococcus should be included in the vaccination Schedule.**								
Strongly disagree	7 (2.0)	16 (1.3)	4.2 (1.7–10.2)		5 (0.8)	18 (3.5)	0.2 (0.1–0.6)	
Disagree	15 (2.3)	12 (3.1)	1.4 (0.7–3.1)		5 (0.8)	22 (4.3)	0.2 (0.1–0.5)	
Neutral	103 (31.9)	125 (28.2)	2.6 (2.0–3.6)	<0.001	150 (24.5)	78 (15.3)	1.8 (1.3–2.4)	<0.001
Agree	182 (24.6)	120 (26.3)	1.2 (0.9–1.6)		187 (30.5)	115 (22.6)	1.5 (1.1–2.0)	
Strongly agree	409 (39.2)	133 (41.2)	0.4 (0.3–0.5)		266 (43.4)	276 (54.2)	0.6 (0.5–0.8)	

UA: University of Alicante; UCAM: Catholic University of Murcia San Antonio; OR is calculated for each response against all the others. *p*-value: calculated for the group, chi-square test; CI: Confidence Interval.

**Table 6:** Knowledge regarding health professionals’ vaccination.

Variable	Mean	SD	*p*-Value
University			
UA	11.14	3.9	0.037
UCAM	10.63	4.17	
Academic year			
Lower	10.63	4.1	0.003
Upper	11.34	3.85	
Gender			
Men	10.39	4.25	0.021
Women	11.09	3.93	
Marital status			
Single	10.98	3.97	NS
Couple	10.31	4.72	
Number of Children			
Yes	10.94	4.0	NS
No	11.41	4.18	
Nationality			
Spain	10.99	3.97	NS
Other	10.15	4.83	
Knowing someone with negative experience			
Yes	11.13	3.96	NS
No	10.89	4.02	
Number of courses with vaccines topic			
No courses	10.13	4.58	0.003
1 or + courses	11.11	3.87	

UA: University of Alicante; UCAM: Catholic University of Murcia San Antonio; SD: Standard deviation. *p*-value: calculated for the group, chi-square test.

## Data Availability

The data presented in this study are available on reasonable request from the corresponding author. The data are not publicly available due to ethical requirements.
